# The single antegrade sling graft: a novel hamstring autograft technique for combined anterior cruciate ligament and anterolateral ligament reconstruction

**DOI:** 10.1007/s00402-024-05697-5

**Published:** 2025-01-15

**Authors:** Ahmed Rabie, Mohamed S. Arafa, Mahmoud Bahloul, Ahmed Abdelbadie

**Affiliations:** 1 Department of Orthopedic Surgery, Faiyum University Hospital, Faiyum, Egypt; 2 Department of Orthopedic Surgery, Suez Canal University Hospital, Kilo 4.5 Ring Road, Ismailia, 41111 Egypt

**Keywords:** ACL graft failure, Anterolateral ligament reconstruction, ACL reconstruction, Combined reconstruction, Hamstring autograft, Soccer, Football, Pivoting sports

## Abstract

**Introduction:**

As a result of increased incidence of anterior cruciate ligament (ACL) injury in young athletes, there is a rise in the indications surgical ACL reconstruction procedures. The value of anterolateral ligament (ALL) reconstruction emerges as a proposed solution to prevent graft failures and improve stability in this high demanding category of patients. The purpose of this study is to present our experience with a novel hamstring auto-grafting technique, the single antegrade sling graft (SASG), for combined reconstruction of both ACL and ALL using autologous gracilis (GR) and semitendinosus (ST) grafts utilizing a single femoral tunnel and double tibial tunnels.

**Materials and methods:**

From January 2020 to December 2021, 21 soccer players were operated utilizing the SASG technique, a modification of the technique of SANTI study group. Inclusion criteria were participating in pivoting sport, high-grade pivot shift examination (Grade 2–3), and evidence of a lateral femoral notch sign or Segond's fracture on preoperative imaging. Patients were assessed for 2 years postoperatively by Lachman’s test for anteroposterior laxity and pivot shift test for rotational laxity. The postoperative outcomes were assessed by Tegner-Lysholm and International knee documentation committee scores. Also, the postoperative complications such as stiffness, infection and graft failure were reported.

**Results:**

21 male soccer players with a mean age 26.4 years were included in this study. After 2 years follow up there was a statistically significant improvement in the both post-operative functional scores, P value < 0.001. Fifteen patients (71.4%) could return to their preoperative sport activity level with no giving-way symptoms. Only one case of graft failure was reported in the follow up.

**Conclusions:**

The single antegrade sling graft (SASG), for combined reconstruction of both ACL and ALL yielded good results in terms of stability and return to sports. The technique is reproducible, and results are comparable to the available published literature.

## Introduction

Anterior cruciate ligament (ACL) tears requiring surgery are very common in athletic young population. It is particularly more common in communities where pivoting sports such as soccer are popular [1]. It was estimated that 283,810 reconstructions were performed annually in the United States with the overall rate of reconstruction increased 22% from 2002 to 2014 [2].

Various techniques for reconstructing the torn ACL were adopted varying from open procedures that are nearly obsolete now, to arthroscopic-assisted ones that represent the gold-standard now in every-day practice. The modern ACL reconstruction techniques provided different grafting techniques and graft fixation methods that managed to restore the anteroposterior laxity. However, the anterolateral rotational stability remained difficult to achieve despite of using more advanced and anatomical procedures [3].

Modern reports suggested that re-enforcement of the anterolateral structures is the key to achieve combined anteroposterior and rotational elements of stability. Recently, the importance of the anterolateral ligament of knee (ALL) gained more research interest [4]. The ALL was first described in 1879, by the French surgeon Paul Segond as a fibrous, resistant, pearly white band that consistently revealed extreme tension when the knee joint is forced into internal rotation [5, 6]. It was not named until 2012; when Vincent et al. [7] termed it as the ALL, despite of being repeatedly described after the initial Segond’s description. Despite of initial denial of the role or even the presence of ALL, a recent cadaveric study proved its presence in almost 80% of our Caucasian population [8]. Further studies on the biomechanical role of the ALL highlighted the importance of its reconstruction to prevent anterolateral instability. The emphasis or even “rediscovery” of the synergistic relation between the intact anterolateral anatomical structures and ACL to prevent anterolateral rotatory instability has led to renewed interest in lateral extra-articular tenodesis procedures and specifically anatomical reconstruction of the ALL [9]. The combined approach in reconstruction of ACL and ALL ligaments represented a hot topic in the orthopaedic literature in the past decade aiming to decrease the rate of the ACL graft ruptures [4, 10, 11].

Modern studies have proposed that the ALL is injured in combination with the torn ACL; suggesting that simultaneous reconstruction of both ligaments will augment the translational stability and add more rotational stability specially in vulnerable patients such as young and high demanding athletes. Multiple reconstruction techniques have been used to repair the ALL aiming to decrease the rate of ACL re-injury after its reconstruction [10–13]. Sonnery-Cottet and his colleagues in 2017 have published their technique of using single hamstring graft for both ACL/ALL with excellent results and reduced ACL graft failure [14]. The later technique was then described thoroughly by Saithna et al. [15]. This research article represents our early experience of a modification of the later technique and our short-term results. We hypothesized that this modified technique will add value of utilizing the whole length of grafted hamstrings, preventing any possible ALL graft damage during insertion, and providing more secured graft fixation with less iliotibial band (ITB) irritation.

## Patients and methods

This case series aimed to present our experience with a novel hamstring auto-grafting technique, the single antegrade sling graft (SASG), for combined reconstruction of both ACL and ALL using autologous gracilis (GR) and semitendinosus (ST) grafts utilizing a single femoral tunnel and double tibial tunnels. The common femoral tunnel was used for fixations of both ACL and ALL. The two separate tibial tunnels; one for fixing each ligament. This technique represents a modification of the technique described by Sonnery-Cottet and his colleagues in 2017 (SANTI study group) [14].

All the patients who had combined ACL and ALL reconstruction and met the inclusion criteria in the period from January 2020 till December 2021 were included in this study. Surgeries were performed in our institute by the first author, patients were followed up for a minimum of 2 years postoperatively.

### Inclusion criteria

Adult male or female with isolated ACL tear only associated with the following

1. Participation in pivoting sports (all our cases were soccer players).
2. Evidence of a high-grade pivot shift (grade 2/3) on examination, the test was carried out by the first author and was confirmed in the operating theater under anesthesia.
3. Evidence of a lateral femoral notch sign (grade 1/2) [16] or Segond's fracture on preoperative imaging.

### Exclusion criteria

1. Adult male or female with ACL tear associated with other ligaments injuries, meniscal injuries or chondral injuries.
2. Pediatric or adolescent patients (with open physis) with ACL tear.
3. Patients with ACL tear and pivot test grade 1.
4. Patients with failed ACL reconstruction.

Oral and written consents were obtained from the all participants before enrollment into the study.

The surgical technique:

## Positioning the patient and bony landmarks

The patient was placed on the operating table in the supine position. A pneumatic tourniquet was applied over the proximal thigh with a leg holder placed lateral to the thigh at the tourniquet level. With the knee flexed about 90°, the bony landmarks were marked; the medial and lateral joint lines, the lateral epicondyle, fibular head, Gerdy’s tubercle, and the tibial insertion of hamstrings.

## Harvesting the hamstring tendons

The autograft was harvested with the conventional method through an oblique incision 6 cm below the medial joint line, 2 cm medial to the tibial tubercle. The tendons could be palpated under the skin by rolling in lean patients. The aponeurosis of the sartorius muscle was incised in line with its fibers and the GR and ST tendons were harvested using an open tendon stripper after resection of the attached vincula tendina. If there was an accessory ST tendon sharing the same insertion [17], it was harvested and used as a graft for ALL reconstruction if more than 3 mm thickness and 10 cm length (there were 3 patients with Type 1–1–2 according to Olewnik Ł et al. [17]. After pulling out the tendons, their tibial insertion was released.

## Arthroscopy

The standard anterolateral and anteromedial arthroscopic portals were used for intra-articular exploration of the menisci and the articular cartilage. The femoral footprint of the ACL was debrided, its middle portion was marked by the electrocautery, and the tibial remnant of the ACL was partially debrided for proprioception preservation.

## The common femoral tunnel preparation (Figs. 1 and 2)

**Fig. 1:**
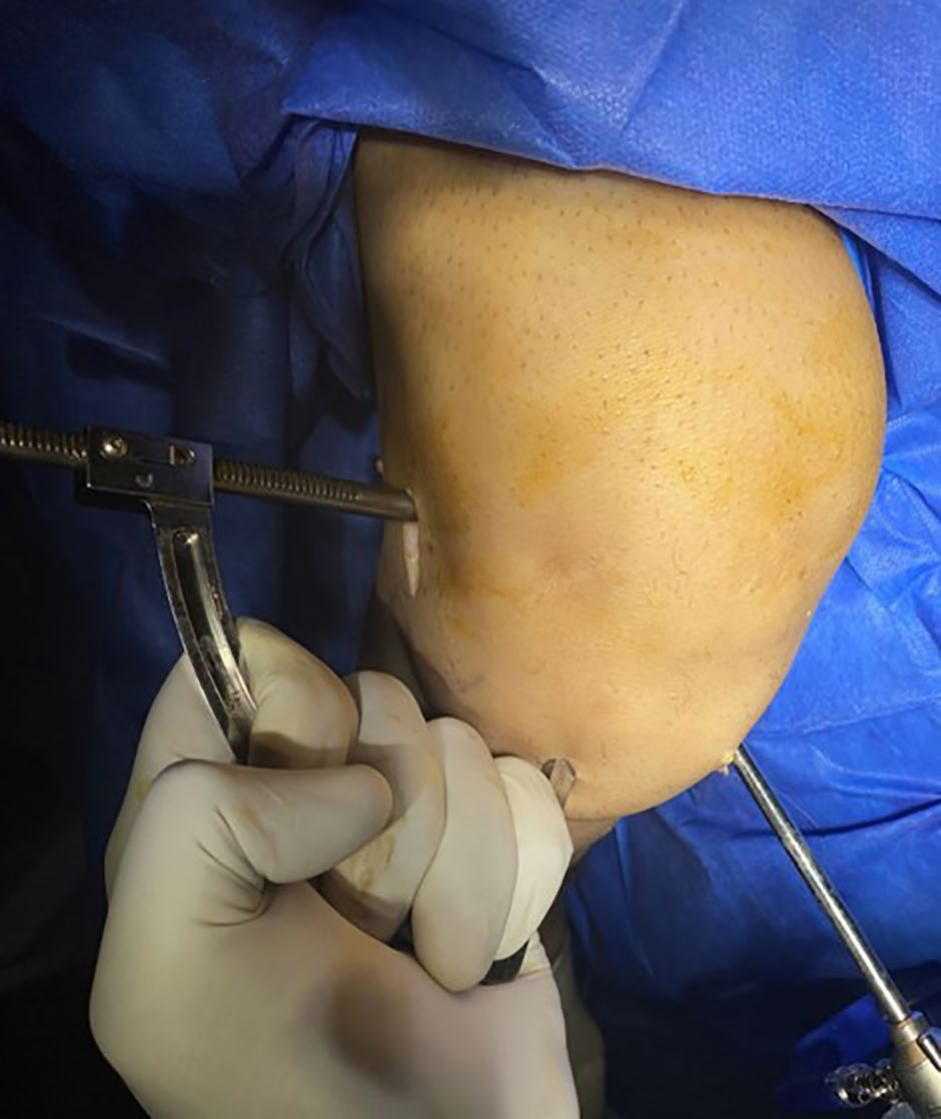
The RT knee intraoperative photo showing the common femoral tunnel preparation with the C- guide through the anterolateral portal and the scope in the anteromedial portal

**Fig. 2:**
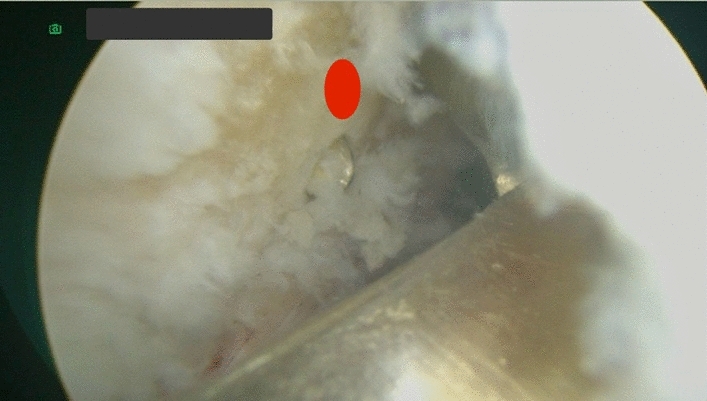
The RT knee arthroscopic view through the anteromedial portal and the guide pen tip in the middle of the femoral ACL footprint

A 3-cm incision of the distal femur through lateral approach was created and centered over the lateral femoral epicondyle. The footprint of the femoral tunnel was marked by splitting the tensor fascia lata just proximal and posterior to the lateral epicondyle. The outside-in C-shaped femoral guide (Arthrex Inc. Naples, Florida, USA) was placed over this mark and intra-articularly through anterolateral portal into the midpoint of the ACL femoral footprint. Then, a complete femoral tunnel was drilled antegrade after passing the guide pin according the measured width of the hamstrings graft. The shaver was used to remove any posterior debris and the intra-articular entrance of the femoral tunnel.

## The tibial tunnel for ACL (Fig. 3)

**Fig. 3:**
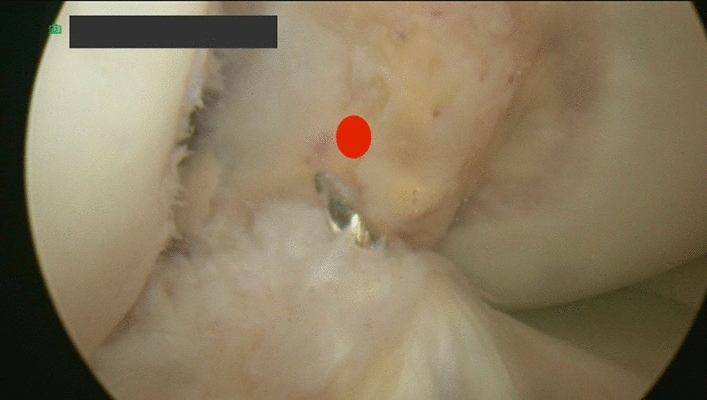
The RT knee arthroscopic view through the anterolateral portal view of the guide pin through the middle of the tibial ACL remnants

Through the hamstrings’ harvesting incision, the ACL tibial tunnel was created above the ACL remnant using the tibial guide set to 55°. The tunnel was drilled according to the thickness of the tibial side of the graft over the guide wire with a low- speed to preserve the tibial remnant of the native ACL.

## The tibial tunnel for ALL

**Fig. 4:**
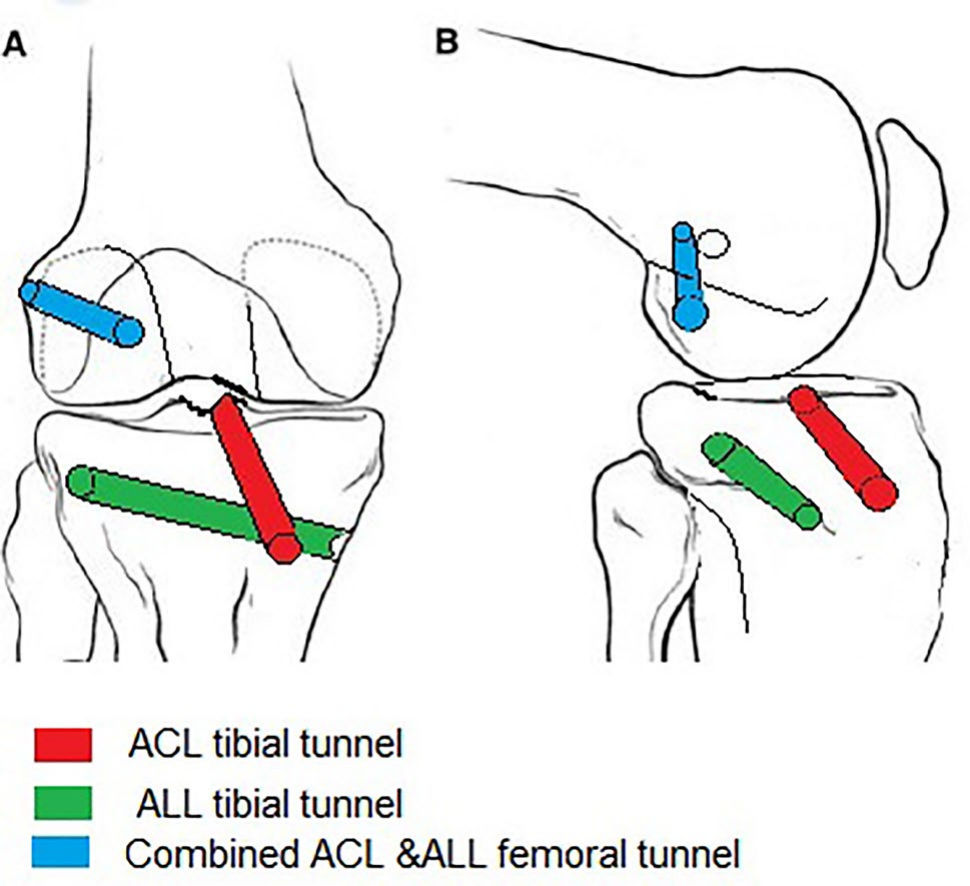
The RT knee schematic drawing showing the tunnels’ orientations. **A** The AP view. **B** The lateral view. 1 The combined ACL & ALL femoral tunnel (blue colored) extends between the intraarticular ACL femoral foot- print on the medial wall of the lateral femoral condyle to a point just above and posterior to the lateral femoral epicondyle. 2 The tibial ALL tunnel (green colored) extends from a midpoint between the Gerdy’s tubercle and the fibular head 1-cm below the lateral joint line towards the anteromedial surface of the tibia at the hamstrings’ harvesting incision. 3 The tibial ACL tunnel (red colored) extends between the tibial ACL foot- print to the anteromedial surface of the tibia at the hamstrings’ harvesting incision

Through a 3-cm incision centered over the midpoint between the Gerdy’s tubercle and the fibular head 1-cm below the lateral joint line, the ALL tibial tunnel was created free-handed with a guide wire then the 4.5-mm drill bit in a downward and forward oblique direction aiming towards the hamstrings’ harvesting incision. First tip, we direct the tunnel distally so as it becomes away from the tibial ACL tunnel. Second tip, we drill the whole length of the tunnel so it will accommodate the whole ALL graft. Schematic drawing showing orientation of the tunnels is shown in (Fig. 4).

## Graft preparation (Fig. 5)

**Fig. 5:**
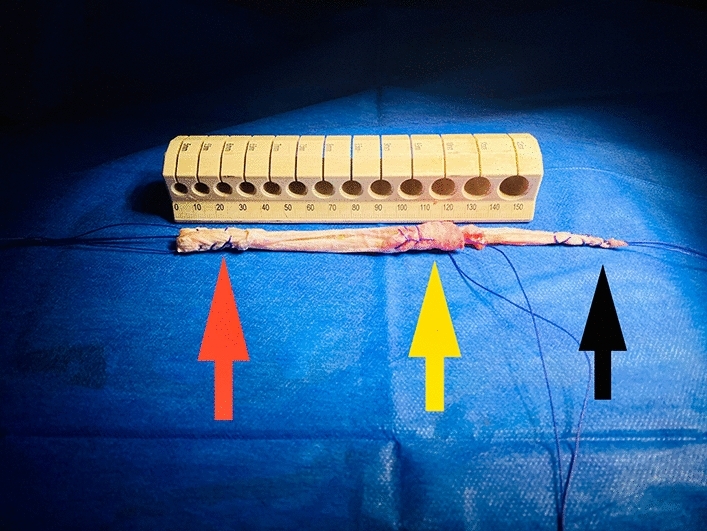
The graft preparation; showing the final the ACL and ALL length. the red arrow is the ACL tibial side, the yellow arrow is the common ACL/ALL femoral side and the black arrow is the ALL tibial side

A Fiber-wire loop was placed through the femoral tunnel, retrieved from the joint and passed through the ACL tibial tunnel until it emerged from the skin at the tibia. It was used to measure the length of the ACL graft, from the lateral edge of femoral tunnel to extra-articular entrance of the tibial tunnel which measured almost around 12 cm (± 1) long. Then, the hamstring tendons were marked, and the tendons were folded upon themselves to create a quadruple graft for the ACL and the extra length of ST was used as a single strand ALL graft. When an accessory ST tendon was found, it was utilized as a second strand for the ALL graft.

## Graft fixation

**Fig. 6:**
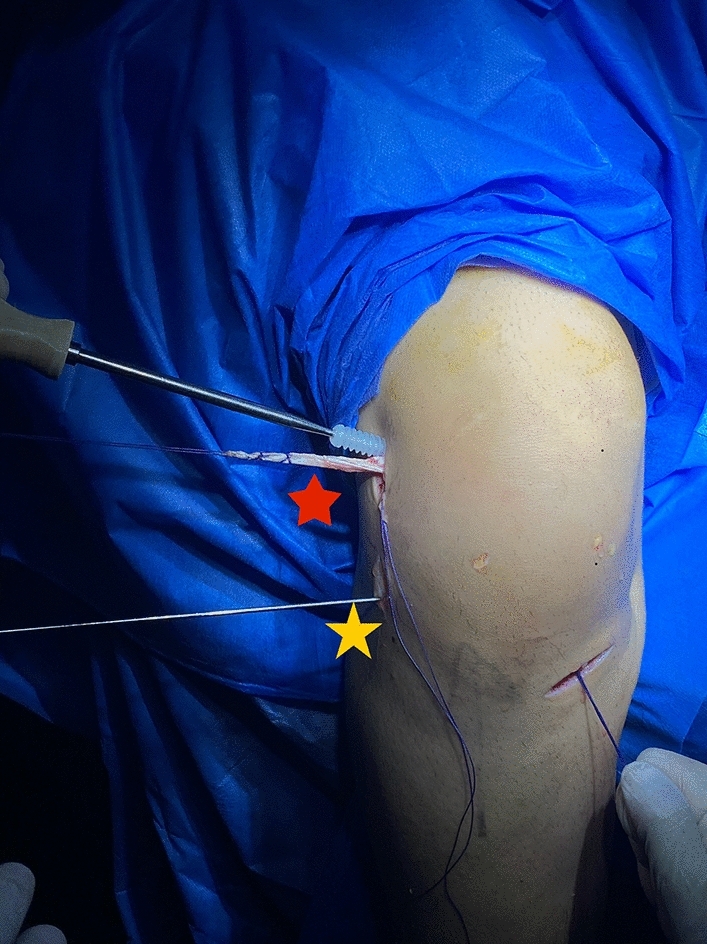
The RT knee intraoperative photo showing the graft passage and fixation at the common femoral tunnel by interference screw with equal tension on the graft ends (femoral and tibial ends during screw insertion to maintain the graft position in the common femoral tunnel). The red star is the ALL graft and the yellow star is the guide pin in the tibial tunnel of the ALL ligament

The graft was passed from outside-in through the femoral tunnel till it reaches the outlet of the tibial tunnel over a stiffened Fiber-wire loop suture, and fixed in outside-in manner first at the femoral tunnel by an absorbable biocomposite interference screw (ComposiTCP^™^ Interference Screw; Zimmer Biomet Orthopedics, Warsaw, Indiana 46,581 USA) with the same size of the femoral tunnel (Fig. 6) while maintaining the traction on the tibial side of the graft. There was no need of additional fixation of the ALL part on the femoral side.

**Fig. 7:**
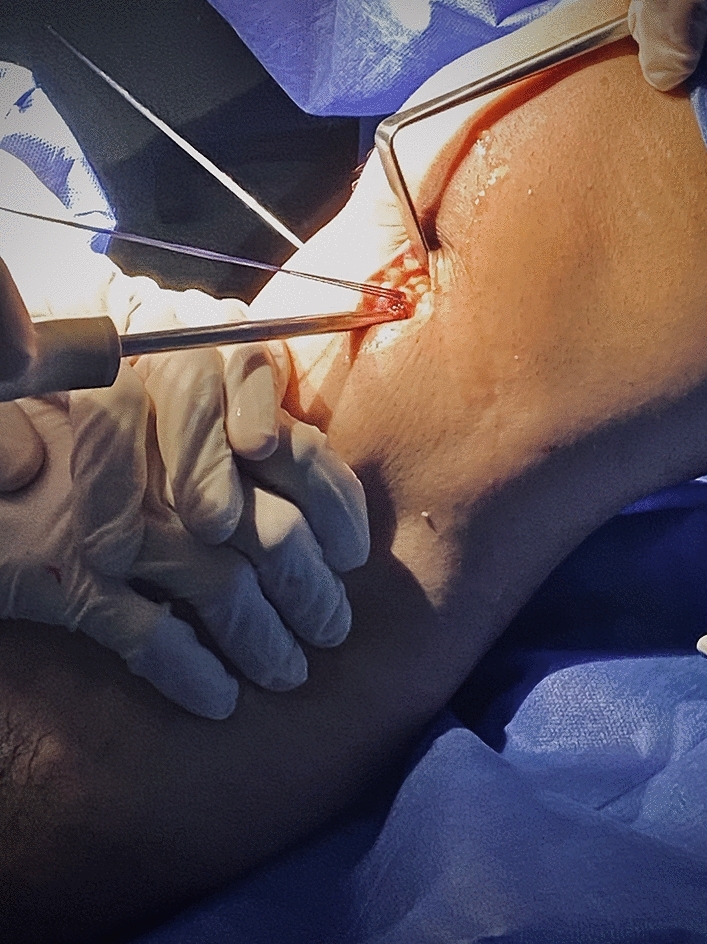
The RT knee intraoperative photo showing the Fixation of the tibial side of the ACL graft by interference screw while applying posterior drawer stress on the proximal tibia

The ACL graft was then fixed to tibial side at 20° flexion and neutral rotation using another absorbable tibial biocomposite interference screw with a size 1 mm larger than the size of the tibial tunnel, with a reverse Lachman maneuver (Fig. 7).

**Fig. 8:**
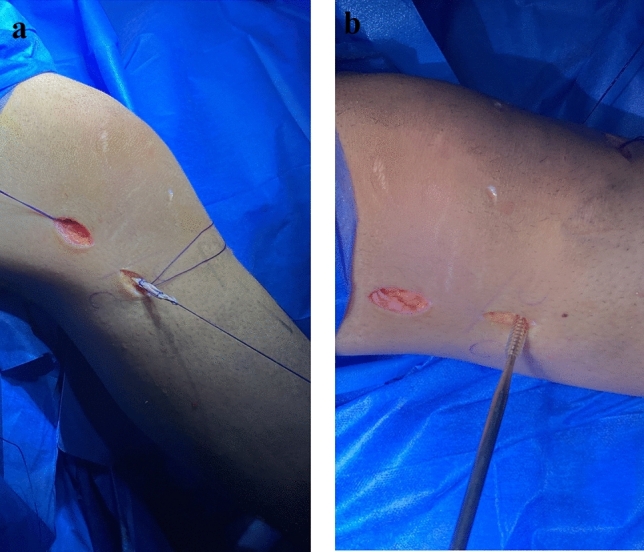
The RT knee intraoperative photo showing: **A** The ALL graft after its advancement distally deep to the ITB. **B** Fixation of the ALL graft in the tibial tunnel by interference screw

Finally, we verified that there was no impingement between the ACL graft and the roof of the notch during knee extension. The ALL graft is advanced under the iliotibial band (ITB) from the proximal to the distal lateral approaches using Halsted clamp and then introduced to the ALL tibial tunnel. And it was attached at neutral extension and rotation using an absorbable tibial biocomposite screw (7 mm diameter, 20-mm length) which is the smallest available diameter (Fig. 8).

## Closure

The incisions are then closed with intradermic absorbable 3.0 sutures, and a compression bandage on the suprapatellar recess is applied. Radiographs are done before the patient is discharged from the outpatient department to assess the bony tunnels.

### Post-operative care and follow-up

The immediate postoperative pivot-shift and Lachman tests were carried out under anaesthesia by the first author. The postoperative follow-up clinical assessments were done on 2 months intervals starting from the 1st month. Assessments were carried out by the second and third authors who did not participate in the surgeries. Weight bearing began as tolerated, with crutches and knee immobilizer for the first 4 weeks post-operatively. Isometric knee extension and active knee range of motion exercise started immediately. The return to sport activities was allowed gradually at 4 months for non-pivoting sports, then at 6 months for pivoting non-contact sports, and finally at 8 to 9 months for pivoting contact sports.

The functional outcomes of the patients have been assessed using Tegner-Lysholm score [18] and International Knee Documentation Committee (IKDC) subjective score [19]. Both scores were reported on each follow-up visit. Early and late complications were documented.

### Statistical analysis

Data were collected and SPSS 17.0 software (SPSS Inc, Chicago, Illinois, USA) was used for data analysis. The association between two qualitative variables was studied by the Fischer’s exact test.

The quantitative variables were assessed using the Mann–Whitney U test. A P-value of < 0.05 was considered the cut-off level for statistical significance.

## Results

During the period between January 2020 to December 2021, 80 patients of combined ACL and ALL reconstruction were operated in our institute. Only 21 patients met the inclusion criteria and 59 patients were excluded (30 patients with meniscal injuries, 10 patients with revision ACL reconstruction, 10 patients refused the technique, and 9 patients didn’t complete the follow up due to travelling abroad).

The remaining 21 patients were followed up for 2 years. All the patients were males with age ranging from 22 to 30 years (mean 26.4) and a BMI ranging from 19 to 26(mean 22.5). There were 7 professional football (soccer) players in the study group, 12 patients were practicing regularly as amateurs, and the remaining 2 were playing as social activity with their friends.

There were 19 patients with lateral femoral condyle notch sign grade 1 that didn’t require any intraoperative intervention and two patients with grade 2 and during the arthroscopy they didn’t require any intervention. There were no patients with a reported preoperative Segond's fracture.

The patients' demographic and intraoperative data are shown in Table 1 and the clinical outcomes are summarized in Table 2. There was a statistically significant improvement in the both post-operative functional scores, P value < 0.001. Fifteen patients (71.4%) could return to their preoperative sport activity level (return to competition) with no giving-way symptoms (neither anteroposterior nor rotational). Five patients could return to noncompetitive sporting activity). Only one patient couldn't return to his previous sport activity due to graft failure during the early follow up.

**Table 1:** The patients' demographic and intraoperative data

Variable	Mean ± SD /#	Range/%
Age (years)	26.4 ± 3	22–30
Time of injury (months)	3.7 ± 1.5	1–6
Side		
Right	15	71.4%
Left	6	28.6%
Thickness of ACL graft		
Femoral	9.7 ± 0.7	9–11 mm
Tibial	8.3 ± 0.5	8–9 mm
ALL graft length	7	6–8 cm
ALL graft thickness	3.5	3–4 mm
Preop pivot shift test		
Grade 2	6	28.6%
Grade 3	15	71.4%
Postop pivot shift test		
Negative	21	100%
Type of graft		
Hamstrings/accessory semitendinosus	3	14.3%
Hamstrings	19	85.7%

**Table 2:** The postoperative clinical outcomes

Variable	Mean ± SD	Range	P-value
The Tegner Lysholm Knee Score			
Pre-op	58 ± 18.1	34–81	< 0.001*
2-year postop	98 ± 2.5	95–100	
Percentage change	77.8 ± 57.6	23.5–179.4	
IKDC			
Pre-op	64.1 ± 8.5	44.8–71.3	< 0.001*
2-year postop	94.7 ± 1.4	93.1–96.6	
Percentage change	56.7 ± 44.6	33.8–113.0	
Return to sports			
Yes	15		71.4%
No	6		28.6%

*Statistically significant

Regarding the post-operative complications, there was one patient who developed superficial infection in the early post-operative period that required frequent dressing and antibiotics only. There was one case graft failure with recurrence of instability during the follow-up period.

## Discussion

The current study aimed to analyze the clinical outcome of a novel hamstring auto-grafting technique, the single antegrade sling graft (SASG), for combined reconstruction of both ACL and ALL with a minimal follow-up of 24 months. 21 soccer players were assessed postoperatively using the pivot-shift test, Tagner-Lysholm as well as the IKDC score. Both scores showed a statistically significant improvement from pre- to postoperatively. 72.4% of the patients could return to their preoperative sport activity level with no instability symptoms. One graft failure was reported at the follow up.

This technique represents a modification of the technique described by Sonnery-Cottet and his colleagues (SANTI study group) in 2017 [14]. Our technique is dependent on the same principles but is different in the following operative steps. In the following lines, we summarized the differences and our proposed rationale behind,

- We have utilized a “Free” hamstring autograft that is separated from the pes anserine insertion, in comparison to an attached ST to its insertion by SANTI study group. We thought that freeing the graft from its insertion allows for utilization of the whole length of the grafted tendons. A recent systematic literature review reported that preserving the graft’s tibial insertion during ACL reconstruction improves the graft's biological incorporation during the early postoperative phases according MRI studies and provides a mechanically stiffer construct. However, equal clinical and functional scores compared to the conventional technique in which the hamstring tendons are detached from their tibial insertion were documented [20].
- The outside-in antegrade introduction of the graft with securing the common femoral part of the 2 grafts (the sling) by a screw gave more reliable estimation of the graft length and prevented any possible damage to the ALL graft during passage through the femoral tunnel in the retrograde passage adopted by the SANTI study group.
- Our ALL part of the graft consists of single or double- strand ST if accessory tendon presents, compared to a single strand GR by the SANTI study group.
- We have drilled 2 tibial tunnels one for each graft, compared to 3 tibial tunnels (2 for ALL and 1 for ACL). The SANTI study group technique described passing the GR grafts through the 2 tibial tunnels and reinforcing the ALL graft by suturing the graft to itself again. We thought that creating a single hole avoids any possible weakness of the lateral tibial head and protects the ITB from irritation by the bulkier ALL graft beneath it.
- We used an interference screw fixation for fixing the ALL graft to the tibia. We believed that it is more reliable and safer with the faster ROM exercises in the early post-operative period, in comparison to re-suturing the graft to itself.

The technique showed a significant improvement in the functional outcome scoring after a minimum of 2 years postoperatively. The return to practice soccer in (15 out of 21) 71.4% of cases without instability. Graft failure was reported only in one case. We found that our inclusion criteria, duration of follow up, and outcome measures were comparable to other studies shown in a recent meta-analysis by Ariel de Lima et al. [21]. On the other hand, the series published by Goncharov et al. [22] showed higher return to sports reaching up to 100% of their 18 patients. The possible cause of difference could be due use of patellar tendon as ACL graft. However, we can argue that this study was carried out on a small sample size [22]. In addition, patellar tendon graft fell out of favor when compared to hamstrings’ graft in the primary reconstruction settings due to higher donor side morbidity [23–25].

In terms of postoperative knee scores, our results are comparable to the published researches that showed marked improvement from the preoperative values [14, 26–28].

The use of interference screw in fixation of both the ACL and the ALL grafts had shown good results in term of fixation strength. Its use proved less graft-tunnel motion, tunnel widening, and graft creep [29]. In addition, it added a solution for the hardware irritation caused by staples that required another operation for surgical removal [30].

We preferred using a single femoral tunnel for both grafts. We claim that it is superior to using two femoral tunnels because it limits the risk of tunnel convergence which may add to the incidence of intraoperative complications. Moreover, it could be less time consuming and cost-effective by decreasing the number of screws or anchors used in grafts fixation [20, 27].

The use of quadruple strand hamstring graft for ACL reconstruction allowed us to reproduce a thicker graft which showed better graft strength and resistance to failure. Small-sized thinner grafts were associated with higher failure rates that represented a major limitation in other studies harvesting a double-stranded hamstring grafts with a diameter around 7 mm [31]. Our preference for using the remainder of ST tendon as ALL graft over the GR was based on the fact that the ST was found longer measuring around 29 cm (± 1 cm) [32], and thicker than the GR tendon [33]. There was no need either to harvest any other graft, or to use an allograft.

A single case of graft failure occurred in this study which is consistent with the reported outcome measures documented in 2 meta-analyses [34, 35].

The limitations in our study are:

1. It is a retrospective study design with no control group with a relatively small number of cases, however, this study can be considered as a technical note. We presented encouraging results of our early cases. Further work on a large case series and randomized controlled trials is needed.
2. No female patients in our cases, due to few numbers of females practicing sports and particularly soccer in our community. As a result, we couldn’t estimate any gender variations.
3. The lack of any biomechanical test to confirm the efficacy of prescribed technique [36]. The objective measurement of stability by machines is not available in our institute.
4. Inefficacy of any preoperative scans to predict the length and diameter of the hamstrings graft.

## Conclusion

The single antegrade sling graft (SASG), for combined reconstruction of both ACL and ALL yielded good results in terms of stability and return to sports. The technique is reproducible, and results are comparable to the available published literature. In addition to our indications, the technique can be useful for vulnerable groups of patients with torn ACL such as younger patients, recurrent, and chronic ACL ruptures.
